# Glycyrrhizic Acid Attenuates Pulmonary Fibrosis of Silicosis by Inhibiting the Interaction between HMGB1 and BRG1 through PI3K/Akt/mTOR Pathway

**DOI:** 10.3390/ijerph19148743

**Published:** 2022-07-18

**Authors:** Zhuoya Niu, Jisong Lin, Changfu Hao, Xiao Xu, Chen Wang, Kai Dai, Xuedan Deng, Meng Deng, Yonghua Guo, Wu Yao

**Affiliations:** 1 Department of Occupational Health and Environmental Health, College of Public Health, Zhengzhou University, Zhengzhou 450001, China; niuzya@163.com (Z.N.); haochangfu@126.com (C.H.); xxiao1995@hotmail.com (X.X.); wang2426500823@163.com (C.W.); daikai0301@163.com (K.D.); dxd690805@163.com (X.D.); dengmeng819025371@163.com (M.D.); yonghuag163@163.com (Y.G.); 2 Department of Nutrition and Food Hygiene, College of Public Health, Zhengzhou University, Zhengzhou 450001, China; linjisong557@163.com

**Keywords:** HMGB1, BRG1, glycyrrhizic acid, silicosis, PI3K/Akt/mTOR signaling

## Abstract

Purpose: High mobility group protein 1 (HMGB1) is a highly conserved DNA-binding nuclear protein that participates in the occurrence and development of silicosis. HMGB1 binds to its specific receptor and activates phosphatidylinositol 3-kinase (PI3K)/protein kinase B, (PKB; Akt)/mammalian target of rapamycin (mTOR) pathway. Brahma-related genes 1 (BRG1; SMARCA4) is the core subunit of SWI/SNF. HMGB1 activates the Akt pathway through BRG1 to promote the proliferation of prostate cancer. Glycyrrhizic acid is a new pharmacological inhibitor of HMGB1, which may inhibit the occurrence and development of silicosis. We speculate that glycyrrhizic acid inhibits the interaction between HMGB1 and BRG1 through the PI3K/Akt/mTOR pathway to affect the progression of silicosis. Methods: We carried out an in vitro study and stimulated A549 with TGF-β1 to establish an epithelial–mesenchymal transition (EMT) model, knocked down the HMGB1 and BRG1 genes in cells, observed the expression of EMT markers, and detected the interaction between HMGB1 and BRG1 by co-immunoprecipitation. In vivo, we injected glycyrrhizic acid into the mouse silicosis model to inhibit the expression of HMGB1. Results: Both HMGB1 and BRG1 were highly expressed in the process of EMT. After knocking down HMGB1 and BRG1, the process of EMT was inhibited through the PI3K/Akt/mTOR pathway, and their expressions were influenced by each other. HMGB1 and BRG1 interact with each other in A549 cells. HMGB1 and BRG1 are also highly expressed in the mouse silicosis model, and glycyrrhizic acid can inhibit the expression of HMGB1/BRG1 through the PI3K/Akt/mTOR pathway. Conclusion: Glycyrrhizic acid can inhibit the interaction between HMGB1 and BRG1 through the PI3K/Akt/mTOR pathway to affect the progression of silicosis.

## 1. Introduction

Pulmonary fibrosis (PF) is a chronic and progressive illness characterized by interstitial fibrosis that leads to irreversible scarring and lung remodeling [1] with a median survival time of 2–4 years after diagnosis [2]. In recent years, a large number of scholars at home and abroad have carried out research on pulmonary fibrosis. However, the specific pathogenesis has not been completely clear. The study of mouse model [3,4] and patients with idiopathic pulmonary fibrosis (IPF) [5] showed that there was a close relationship between the epithelial–mesenchymal transition (EMT) and the process of pulmonary fibrosis. EMT is a biological process in which epithelial cells transform into cells with a mesenchymal phenotype, which is characterized by decreased expression of epithelial markers and increased expression of mesenchymal markers. Through EMT, epithelial cells undergo significant phenotypic changes, from tightly connected paving stone-like epithelial cells to elongated spindles to form fibroblast-like morphology [6].

High mobility group protein 1 (HMGB1) is a highly conserved non-histone chromosome protein in the nucleus [7]. As a DNA chaperone, it participates in chromatin remodeling, nuclear transcription, replication, DNA repair, and nucleosome assembly. At the same time, HMGB1 can be secreted or released out of the cell as a damage-related model molecule that binds to its receptor to participate in inflammation, cell differentiation, cell migration, angiogenesis and tumor metastasis, drug resistance, and so on [8,9]. In addition, HMGB1 can induce EMT in colorectal cancer, liver cancer, non-small cell lung cancer [10,11], and other tumors, and promote cell migration and invasion.

Brahma-related genes 1 (BRG1) is the core subunit of SWI/SNF, an ATP-dependent chromatin remodeling complex. Some studies have found that Zinc finger E-box binding homeobox 1 (ZEB1) interacts with BRG1 to inhibit the E-cadherin (E-cad) promoter [12]. ZEB1 and BRG1 have been reported to co-localize in E-cadherin-negative cells, normal colonic stroma, and dedifferentiated epithelial cells at the infiltrating front of colorectal cancer. Other studies have shown that HMGB1 promotes the proliferation of prostate cancer by activating protein kinase B (PKB/Akt) pathway through BRG1. [13] However, it has not been studied in pulmonary epithelial cells and silicosis.

Glycyrrhizic acid is an HMGB1 inhibitor [14], which is approved by FDA to treat hepatitis. Some studies have shown that HMGB1 exists in the early stage of pulmonary inflammation and late fibrosis, and glycyrrhizic acid inhibits the expression of HMGB1, which limits the progress of pulmonary toxicity [15]. Previous studies have shown that glycyrrhizin inhibits HMGB1 and thus inhibits EMT by blocking the downstream Smad2/3 signal pathway [16], but there is no study on glycyrrhizic acid in silica-induced silicosis in mice. We hypothesized that HMGB1 and BRG1 interact with each other in lung epithelial cells, and glycyrrhizic acid impairs PF by inhibiting their interaction through the phosphatidylinositol 3-kinase (PI3K)/Akt/mammalian target of rapamycin (mTOR) pathway. To test this hypothesis, we constructed a silica-induced silicosis model and TGF-β1-induced EMT model, respectively, to determine the unknown relationship between HMGB1 and BRG1 and the role of glycyrrhizic acid in EMT-mediated PF.

## 2. Materials and Methods

### 2.1. Animal Model

Male C57BL/6N mice (4–5 weeks old, 20–22 g) were obtained from Beijing Vital River Laboratory Animal Technology Company (Beijing, China). Mice were kept in ventilated cages at 25 °C, 12 h light/dark cycle, 45–55% humidity, and under specific pathogen-free conditions. Mice were randomly divided into the following groups (*n* = 8–9 per group): a control group, a silica group, a solvent control group, and a glycyrrhizic acid group. Under isoflurane anesthesia, the control group was injected with 100 μL normal saline, and other groups of mice were intratracheally instilled with 100 μL sterile SiO_2_ dust suspension (50 mg/mL). The mice in glycyrrhizic acid group were administered glycyrrhizic by intraperitoneal injection daily with 50 mg/kg and the mice in the solvent control group were injected with 100 μL solvent (2%DMSO + 98%saline) every day. After receiving silica for 28 days, all experimental mice were killed by pentobarbital sodium anesthesia. The left lung was fixed and used to make pathological sections, and the right lung was used for follow-up experiments. This study was performed in line with the principles of the Declaration of Helsinki. All animal experiments were approved by the Animal Research Ethics Committee of Zhengzhou University, and the approval number (ZZUIRB 2021-120) was identified.

### 2.2. Cell Lines, Small Interference RNA and Transfection

Human non-small cell lung cancer cells (A549) were obtained from the cell bank of the Chinese Academy of Sciences. The cells were cultured in 1640 medium (Solarbio, Beijing, China) containing 10% fetal bovine serum (FBS; Invigentech, Brazil), and then cultured in a 5% CO_2_ humidified incubator at 37 °C.The HMGB1 small interfering RNA (siHMGB1), BRG1 small interfering RNA (siBRG1), and negative control siRNA (siNC) were synthesized according to human-specific sequences by GenePharma (Shanghai, China). The siRNA sequences are listed as follows: 

HMGB1-siRNA: sense 5′-CCCGUUAUGAAAGAGAAAUTT-3′ and antisense 5′-AUUUCUCUUUCAUAACGGGTT-3′; 

BRG1-siRNA: sense 5′-CUCAGAUCAUGGCCUACAATT-3′ and antisense 5′-UUGUAGGCCAUGAUCUGAGTT-3′. 

They were transfected into A549 cells with Lipofectamine iMax according to the manufacturer’s instructions. 

In addition, after A549 cells were treated with different concentrations of glycyrrhizic acid for two hours, the culture medium containing TGF-β1 was changed, and then the EMT markers were detected.

### 2.3. Histologic Analysis

After 28 days of dust exposure, the mice were killed, their lungs were removed and fixed overnight with 4% paraformaldehyde. The lung tissue was embedded in paraffin, sectioned at 5 μm, and stained with hematoxylin-eosin (HE) and Masson’s trichrome (Masson).

### 2.4. Immunohistochemical Analysis

Tissue sections were dewaxed and rehydrated with graded ethanol. After antigen repair, the sections were incubated with normal goat serum diluted by PBS and then incubated with the primary antibody working solution and the secondary antibody working solution respectively. After adding antibodies such as ABC complex and rinsing with phosphate buffer, the sections were stained with DAB and re-stained with hematoxylin. After dehydration, the slices were air-dried and observed under a microscope. The results were analyzed by ImageJ software.

### 2.5. Protein Extraction and Western Blot Analysis

Animal and cell samples were added with cleavage buffers (Dingguo, Beijing, China) (1% protease inhibitor and 2% phosphatase inhibitor) on the ice. Animal samples are ground twice in a grinder (Servicebio, Wuhan, China) for one minute each time. After that, animal samples and cell samples were treated with a high-intensity ultrasound processor (Scientz, Ningbo, China) in lysis buffer 3 times. The sample was centrifuged at 4 °C at a temperature of 12,000× *g* for 10 min. Finally, the supernatant was collected and the protein concentration was determined according to the manufacturer’s instructions with BCA kit (BOSTER, Wuhan, China).

Proteins in cells and tissues were separated by SDS-PAGE and transferred to PVDF membrane. Western blot follows the method discussed in a recent paper [17]. Western blot uses the following primary antibodies: anti-HMGB1 (Proteintech, 10829-1-AP, 1:1500), anti-BRG1 (abcam, ab110641, 1:15,000), anti-N-cad (Servicebio, GB111009, 1:1000) anti-Vimentin (Servicebio, GB11192, 1:1000) anti-SMA (Servicebio, GB13044, 1:1500), anti-E-cad (Cell Signaling Technology, 3195, 1:1000), anti-PI3K (Proteintech, 20584-1-AP, 1:1000), anti-Akt (Proteintech, 60203-2-Ig, 1:10,000), anti-mTOR (Proteintech, 66888-1-Ig, 1:10,000), anti-phospho-PI3K (Cell Signaling Technology, 4228T, 1:1000), anti-phospho-Akt (Cell Signaling Technology, 4060, 1:2000) anti-phospho-mTOR (Cell Signaling Technology, 5536T, 1:1000) and anti-GAPDH (Proteintech, 60004-1-lg, 1:20,000). Specific information is provided in the Supplementary Materials.

### 2.6. RNA Extraction and Quantitative real-Time PCR (qRT-PCR) Assays 

According to the instructions, RNA was extracted from cells or animal tissues with RNAiso Plus reagent (Takara, Kusatsu City, Shiga Prefecture, Japan). The quality and quantity of RNA were verified by NanoDrop 2000 spectrophotometer (Thermo Science, Waltham, MA, USA). RT-qPCR was carried out on a Quant-Studio 7 Flex Rcal-Time PCR System (Thermo Fisher AppliedBiosystems, Waltham, MA, USA) using SYBR Green PCR kit (Takara). The primers used in this study were designed and provided by Sangon Biotech. The test was repeated three times. The data were shown as mean ± standard deviation (SD), and the data were analyzed by 2^−ΔΔCt^ method.

### 2.7. Cell Scratch Assay

A549 cells were inoculated in 6-well plates at a density of 5 × 10^5^ per well and grew to 70% and 80%. A cross line was drawn on the back of the six-hole plate and use a 10-microliter gun was used to evenly draw the cells at the bottom of the hole along the line. The cell fragments were washed with phosphate buffer solution 3 times. The cells were then treated with TGF-β1 or rhHMGB1, and the wounds in the scraped area were monitored and captured by an inverted microscope (OLYMPUS) at a specified time point. The distance from both sides of the scratch was quantified in three different areas within the same scratch.

### 2.8. Co-Immunoprecipitation

In short, an appropriate amount of precooled IP cell lysate was added to the cell petri dish, and then A549 cell suspension was collected. A total of 1.0 μg IgG and 20 μL protein A/G beads were added to the supernatant of negative control (IgG) protein, and 20 μL protein A/G beads were directly added to the experimental group. The supernatants were centrifuged after being shaken and incubated at 4 °C for 1 h. Add 1–10 μL (0.2–2 μg) antibody and incubate for 4 °C overnight. After further incubation with 80 μL A/G pellets at 4 °C for 2 h, the immunoprecipitate was washed with 1 mL precooled IP lysate. After the last washing, the supernatant was absorbed as much as possible, then 80 μL 1 × reduced sample buffer was added and boiled for 10 min of denaturalization. The supernatant was detected by immunoblotting with anti-HMGB1 (10829-1-AP, proteintech) and anti-BRG1 (21634-1-AP, proteintech) antibodies.

### 2.9. String Database

We predicted the proteins that may interact with HMGB1 through the String database (https://cn.string-db.org/, accessed on 10 April 2022), and analyzed and compared the results.

### 2.10. Statistical Analysis

All the experimental data were statistically analyzed by SPSS21.0 (IBM, Armonk, NY, USA). The measurement data were compared by two independent sample *t*-test and single factor analysis of variance (α = 0.05). The bar chart is drawn by GraphPad8.0 software (GraphPad Software, San Diego, CA, USA).

## 3. Results

### 3.1. Both HMGB1 and BRG1 Are Elevated in TGF-β1-Induced EMT

**Figure 1:**
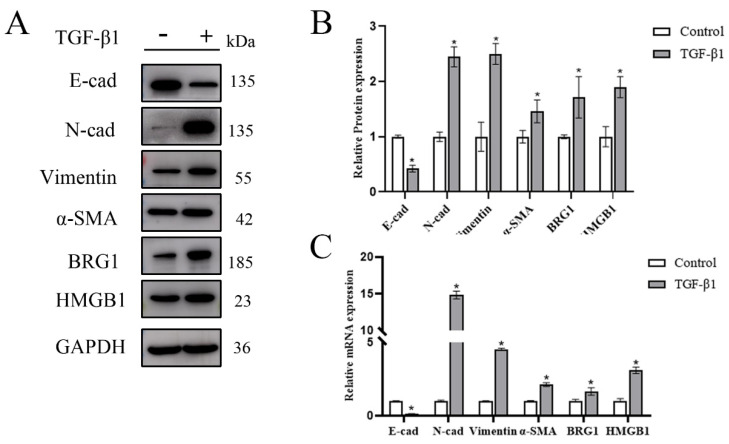
In the EMT model stimulated by TGF-β1, the expression of HMGB1 and BRG1 increased. (**A**,**C**) After 48 h of culture in the medium containing 5 ng/mL TGF-β1, E-cad, N-cad, Vimentin, SMA, HMGB1, BRG1, and GAPDH were detected by Western blotting and RT-qPCR. (**B**) Quantification. GAPDH was used as an internal control. Shown are the averages of three independent experiments. *: *p* < 0.05 compared with the control cells.

Previous studies have shown that HMGB1 increases in EMT models of A549 and BEAS 2B cells. In order to further verify whether HMGB1 and BRG1 play a role in EMT, we stimulated A549 cells with TGF-β1 to establish an EMT model, and Western blot and RT-qPCR were used to analyze the changes of HMGB1 and BRG1 during EMT. Figure 1A–C shows that the gene expression and protein expression of HMGB1 and BRG1 are increased during EMT.

### 3.2. Silencing the Expression of HMGB1 Can Inhibit the EMT Process of A549 Cells

**Figure 2:**
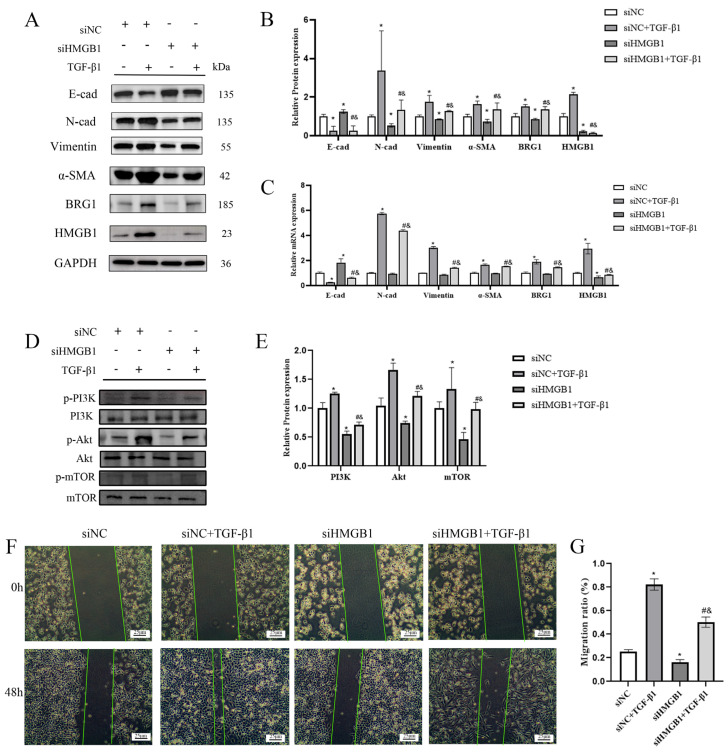
Knocking down HMGB1 can slow down the EMT process through the PI3K/Akt/mTOR pathway. (**A**,**C**) After silencing HMGB1, cultured in normal medium or medium containing 5 ng/mL TGF-β1 for 48 h. The expression of E-cad, N-cad, Vimentin, α-SMA, HMGB1, BRG1, and GAPDH were detected by Western blotting and RT-qPCR. (**B**) Quantification. (**D**) After silencing HMGB1, the expression of PI3K, p-PI3K, Akt, p-Akt, mTOR, and p-mTOR were detected by Western blotting. (**E**) quantification. (**F**) A scratch test was carried out after knocking down HMGB1. (**G**) Quantification. GAPDH was used as an internal control *: *p* < 0.05 compared with the control group. #: *p* < 0.05, compared with the siHMGB1 group; &: *p* < 0.05, compared with the siHMGB1 + TGF-β1 group.

We knocked down the expression of HMGB1 in A549 cells and observed its effect on EMT progression. Figure 2A–C showed that silencing HMGB1 affected the expression of EMT markers, such as the increased expression of E-cad and the decreased expression of N-cad, Vimentin and α-SMA, which showed that silencing HMGB1 inhibited the occurrence and development of EMT process. Further research has shown that silencing HMGB1 can also inhibit the expression of PI3K/Akt/mTOR pathway and the ability of cell migration. In addition, we also observed that after silencing HMGB1, the expression of BRG1 was also inhibited (Figure 2D–G). We speculate that there is an interaction between HMGB1 and BRG1 in A549 cells.

### 3.3. HMGB1 Can Interact with BRG1 in A549 Cells

**Figure 3:**
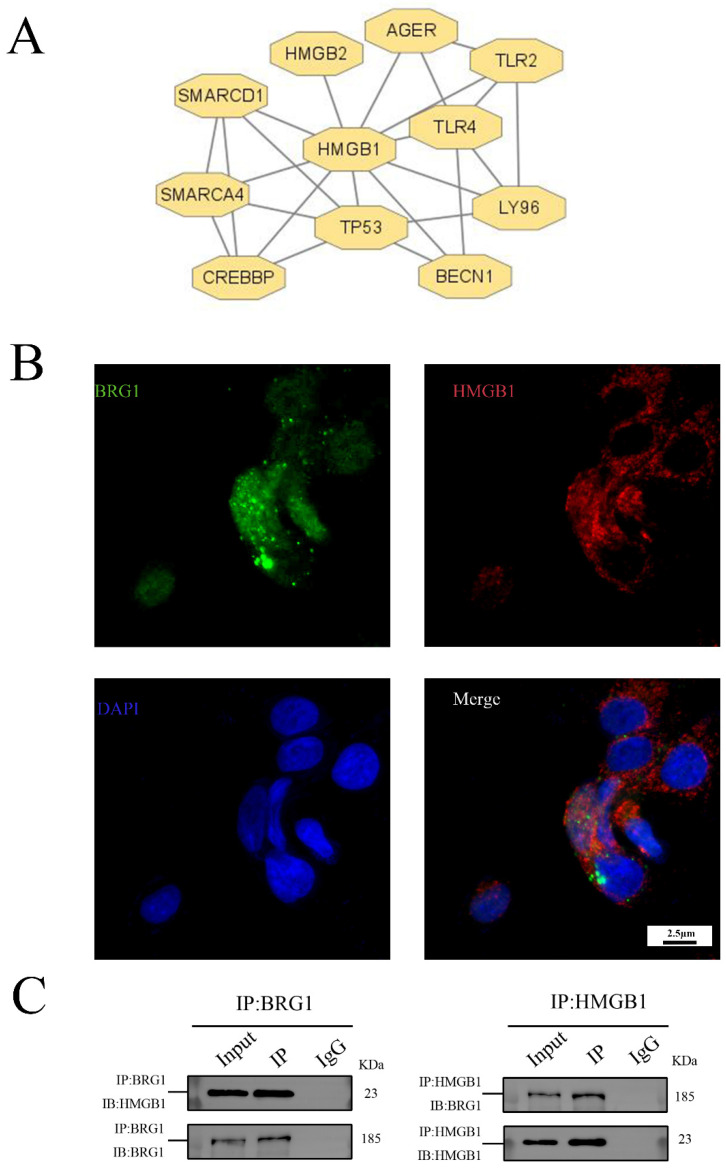
HMGB1 can interact with BRG1 in A549 cells. (**A**) String system was used to predict the proteins interacting with HMGB1. (**B**) Results of immunofluorescence localization of HMGB1 and BRG1 in A549 cells (×630). (**C**) Co-immunoprecipitation verification of the interaction between HMGB1 and BRG1.

We use the string database to predict, as shown in Figure 3A, the proteins that interact with HMGB1 include BRG1. In order to verify this prediction, we carried out immunofluorescence assay. As shown in Figure 3B, there is an overlap between HMGB1 and BRG1 in A549 cells, that is, there is a co-localization relationship between them. In addition, the results of co-immunoprecipitation are shown in Figure 3C. As expected, HMGB1 precipitates with BRG1 in the interchange common IP of the beads bound to BRG1. Our results confirm our hypothesis that HMGB1 and BRG1 can interact with each other in A549 cells.

### 3.4. Silencing BRG1 Can Inhibit EMT Process, While up-Regulation of HMGB1 Can Reverse This Inhibition

**Figure 4:**
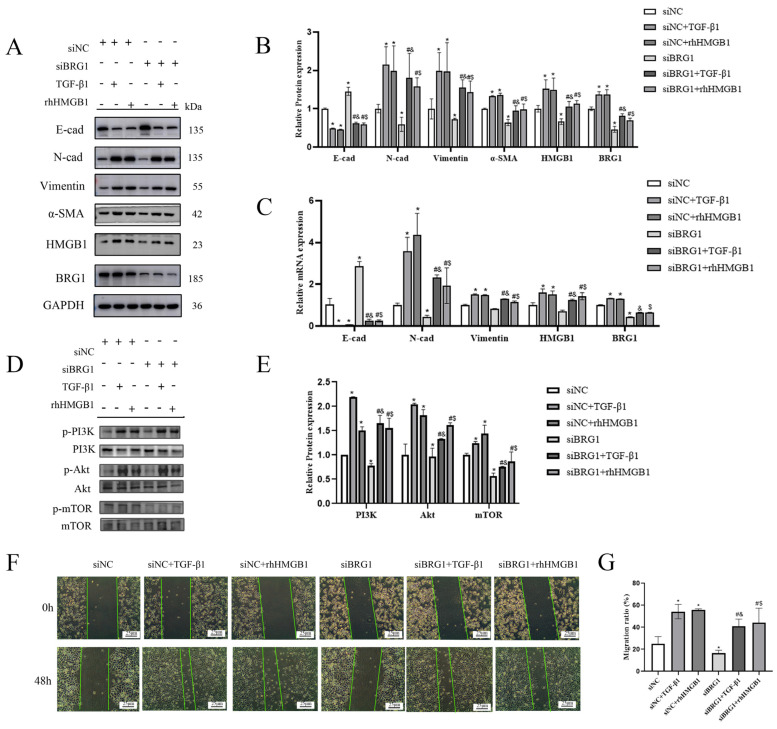
Knocking down BRG1 can slow down the EMT process through PI3K/Akt/mTOR pathway. (**A**,**C**) After silencing BRG1, cultured in normal medium or medium containing 5 ng/mL TGF-β1 or 4 ng/mL rhHMGB1 for 48 h. The expression of E-cad, N-cad, Vimentin, α- SMA, HMGB1, BRG1, and GAPDH were detected by Western blotting and RT-qPCR. (**B**) quantification. (**D**) After silencing BRG1, the expression of PI3K, p-PI3K, Akt, p-Akt, mTOR, p-mTOR were detected by Western blotting. (**E**) Quantification. (**F**) A scratch test was carried out after silencing BRG1. (**G**) Quantification. GAPDH was used as an internal control *: *p* < 0.05 compared with the control group. #: *p* < 0.05, compared with the siBRG1 group; &: *p* < 0.05, compared with the siBRG1 + TGF-β1 group. $: *p* < 0.05, compared with the siBRG1 + rhHMGB1 group.

In order to further verify the mechanism of HMGB1 and BRG1 in the EMT process of A549 cells, we silenced the expression of BRG1 and stimulated A549 cells with rhHMGB1. Figure 4A,E shows that silencing BRG1 inhibited the EMT process and inhibited the expression of HMGB1, and the PI3K/Akt/mTOR pathway was also inhibited. The use of exogenous HMGB1 promotes the process of EMT and the expression of BRG1 as well as PI3K/Akt/mTOR pathway. Silencing BRG1 inhibits cell migration, while up-regulation of HMGB1 reverses this inhibition. (Figure 4F,G) These results indicate that HMGB1 can interact with BRG1 and affect the EMT process of A549 cells through PI3K/Akt/mTOR pathway.

### 3.5. Different Concentrations of Glycyrrhizic Acid Could Inhibit the EMT-Like Changes Caused by TGF-β1

**Figure 5:**
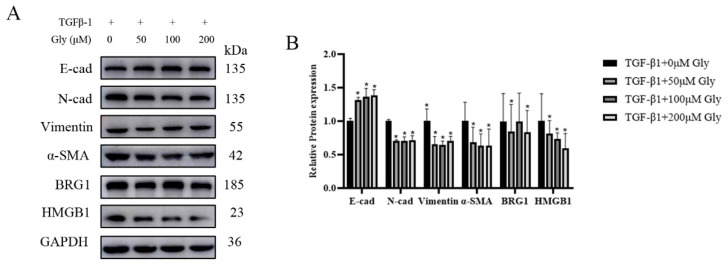
Different concentrations of glycyrrhizic acid could inhibit the EMT-like changes caused by TGF-β1. After A549 cells were treated with different concentrations of glycyrrhizic (0, 50, 100, 200 μM) acid for two hours, the culture medium containing 5 ng/mL TGF-β1 was changed, and then the EMT markers were detected. (**A**) The expression of E-cad, N-cad, Vimentin, α-SMA, HMGB1, BRG1, and GAPDH were detected by Western blotting. (**B**) Quantification. Shown are the averages of three independent experiments. GAPDH was used as an internal control *: *p* < 0.05 compared with the TGF-β1+0 μM Gly group.

Glycyrrhizic acid, as known as a natural HMGB1 inhibitor, plays an important role in acute lung injury and TGF-β1 induced EMT. After A549 cells were treated with different concentrations of glycyrrhizic acid for two hours, the culture medium containing TGF-β1 was changed. The determination of protein expression in cells showed that after glycyrrhizic acid treatment, the expression level of E-cad was increased at different degrees, while the expression levels of N-cad, Vimentin, and α-SMA were decreased at different degrees. The EMT process was inhibited and the expression levels of HMGB1 and BRG1 were decreased at different degrees (Figure 5A,B).

### 3.6. Protective Effect of Glycyrrhizic Acid on Silica-Induced Silicosis in Mice

**Figure 6:**
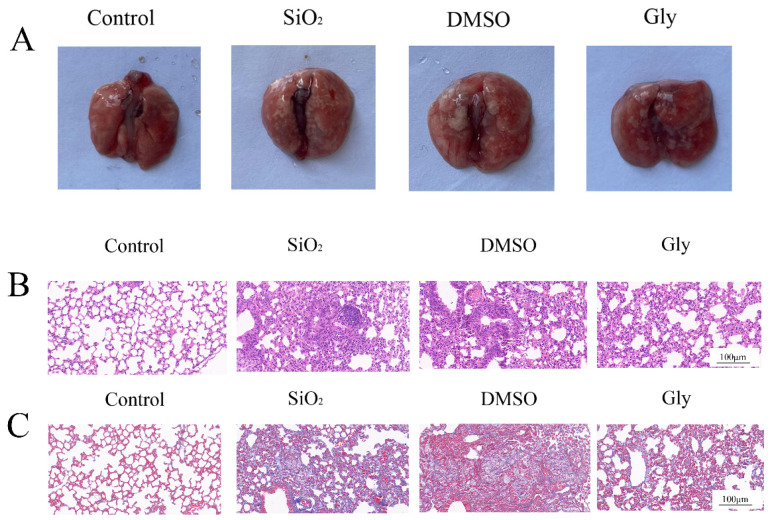
Protective effect of glycyrrhizic acid on pulmonary fibrosis in mice. (**A**) Morphological observation of the lung. (**B**) Hematoxylin–eosin (HE) of lung tissue sections in mice. (×200). Scale bar = 100 μm. (**C**) Masson staining of lung tissue sections in mice. (×200). Scale bar = 100 μm.

We speculate that glycyrrhizic acid can also inhibit the expression of HMGB1 in silica-induced pulmonary fibrosis. Therefore, we established a mouse silicosis model and injected a certain concentration of glycyrrhizic acid into the mice every day. From the results of the lung morphology and pathological section, the fibrosis process was significantly inhibited after injection of glycyrrhizic acid (Figure 6A–C). Therefore, glycyrrhizic acid plays a protective role in the process of silicosis in mice.

### 3.7. Glycyrrhizic Acid Can Alleviate the EMT Process in Mice Silicosis

**Figure 7:**
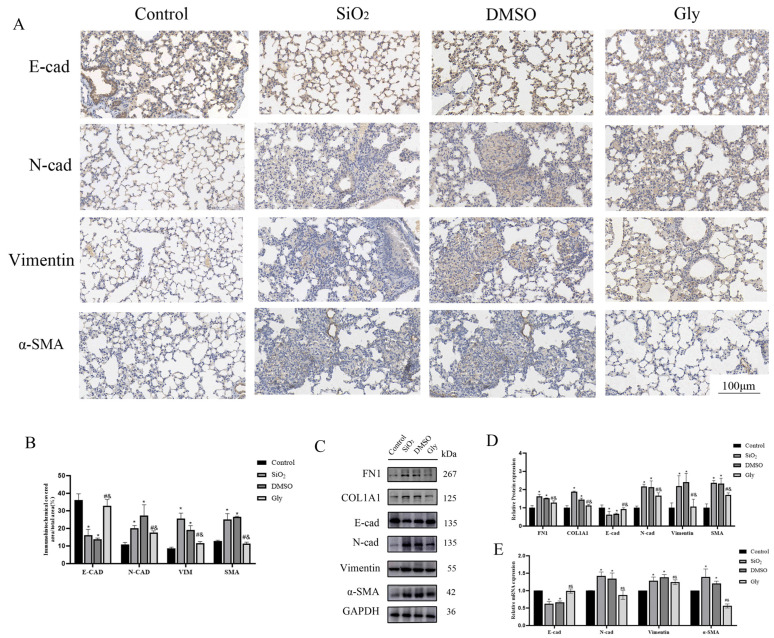
Glycyrrhizic acid inhibits the process of EMT. (**A**) IHC staining showing E-cad, N-cad, Vimentin, and SMA expression in these four groups respectively. (×200). Scale bar = 100 μm. Blue indicates the location of the nucleus and brown indicates the expression of specific proteins. (**B**) Quantification. (**C**,**E**) The expression of FN-1, COL-1, E-cad, N-cad, Vimentin, α-SMA, and GAPDH were detected by Western blotting and RT-qPCR. (**D**) Quantification. Shown are the averages of three independent experiments. GAPDH was used as an internal control *: *p* < 0.05 compared with the control group; #: *p* < 0.05, compared with the SiO_2_ group; &: *p* < 0.05, compared with the solvent control group.

To explore whether glycyrrhizic acid affects the progression of silicosis by inhibiting the HMGB1-promoted EMT process, we performed further experiments. In the immunohistochemical results, blue indicates the location of the nucleus and brown indicates the expression of specific proteins. Immunohistochemical, Western blot, and PCR results showed that glycyrrhizic acid promoted the expression of EMT marker E-cad and inhibited the expression of N-cad, Vimentin, and α-SMA (Figure 7A–D), suggesting that glycyrrhizic acid can inhibit the EMT process in silicosis. 

### 3.8. Glycyrrhizic Acid Inhibits the Interaction between HMGB1 and BRG1 through PI3K/Akt/mTOR Pathway

**Figure 8:**
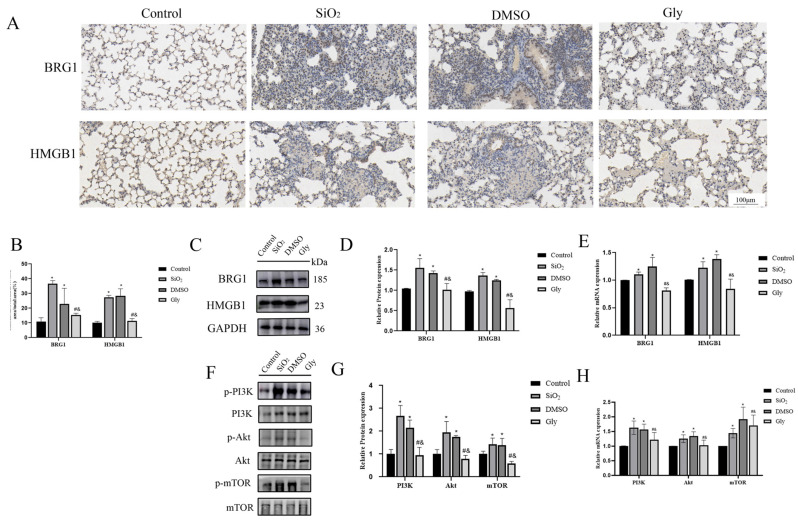
Glycyrrhizic acid inhibits the expression of HMGB1 and BRG1 through PI3K/Akt/mTOR pathway. (**A**) IHC staining showing HMGB1 and BRG1 expression in these four groups respectively. ×200. Scale bar = 100 μm. Blue indicates the location of the nucleus and brown indicates the expression of specific proteins. (**B**) Quantification. (**C**,**E**) The expression of HMGB1, BRG1, and GAPDH were detected by Western blotting and RT-qPCR. (**D**) Quantification. (**F**,**H**) PI3K, p-PI3K, Akt, p-Akt, mTOR, and p-mTOR expression in mouse lung tissue were detected by Western blotting and RT-qPCR. (**G**) Quantification. Shown are the averages of three independent experiments. GAPDH was used as an internal control *: *p* < 0.05 compared with the control group; #: *p* < 0.05, compared with the SiO_2_ group; &: *p* < 0.05, compared with the solvent control group.

Our results show that HMGB1 and BRG1 can co-act on the PI3K/Akt/mTOR pathway to affect the process of EMT in cells, so can glycyrrhizic acid affect the interaction between HMGB1 and BRG1 through the same pathway in animals? We carried out Western blot and RT-qPCR experiments. The results confirmed our conjecture. The expression of BRG1 in lungs of mice injected with glycyrrhizin was also lower than that of the model and the solvent control group, indicating that glycyrrhizin can affect the interaction between HMGB1 and BRG1. (Figure 8A–D). The PI3K/Akt/mTOR pathway was significantly inhibited in mice injected with glycyrrhizic acid (Figure 8E–G). Therefore, we infer that glycyrrhizic acid inhibits the interaction between HMGB1 and BRG1 through the PI3K/Akt/mTOR pathway.

## 4. Discussion

The high mortality rate of 45% from fibrotic diseases in developed countries places a significant burden on public health systems [18]. Silicosis is an important disease in fibrotic diseases. It is mainly caused by the excessive deposition of extracellular matrix, which leads to the destruction of the normal structure and function of alveoli. At present, the research on the pathogenesis of silicosis is mainly focused on the epithelial cell injury, TGF-β system, and EMT process. EMT is a pathophysiological process in which epithelial cells lose part of their epithelial features and acquire stromal features. This process often occurs in three states, namely, development, cancer, and fibrosis, in which tissue damage and remodeling destroys the homeostasis of normal tissue [19]. In rat and mouse silicosis models, the expression of E-cad decreased significantly, while the expression of Vimentin and α-SMA increased significantly [20,21]. It is also confirmed that EMT is involved in the development of silicotic fibrosis in vitro models associated with silicosis [22]. Direct stimulation of TGF-β1 can induce the EMT process in A549 cells and play a corresponding role in the progression of pulmonary fibrosis. Therefore, as stated in the previous study [16,23], we used the medium containing 5 ng/mL TGF-β1 to establish an in vitro EMT model. The results proved that the in vitro EMT model was successfully constructed, but the mechanism of EMT is not clear.

Some studies have found that HMGB1 is an important molecule involved in the EMT process. HMGB1 can secrete or release cells as injury-related molecules, and bind with its receptors to participate in inflammation, cell differentiation, cell migration, angiogenesis, tumor metastasis, drug resistance, and other processes. It plays an important role in inflammatory response, vascular remodeling, and fibrosis in many lung diseases [24]. In the in vitro model of EMT established in this study, the expression of HMGB1 in A549 cells stimulated by TGF-β1 increased, which was consistent with previous studies [25]. After silencing the expression of HMGB1 in A549 cells with siRNA, the process of EMT was inhibited, and the migration ability of cells was also inhibited. HMGB1 plays an important role in the process of EMT. After silencing the expression of the HMGB1 gene, the expression of BRG1 was also inhibited, so it was speculated that there was a certain relationship between HMGB1 and BRG1 in A549 cells. Some studies have pointed out that HMGB1 and BRG1 co-locate and interact with each other in prostate cancer cells, and HMGB1 can positively regulate the expression of BRG1 [13]. The prediction results of the String database show that there may be an interaction between BRG1 and HMGB1. Therefore, in this study, the immunofluorescence experiment was carried out, and it was observed that HMGB1 and BRG1 were co-located in A549 cells, and then two-way immunoprecipitation experiments showed that BRG1 and HMGB1 could co-precipitate, which indicated that there was interaction between HMGB1 and BRG1 in A549 cells. In order to verify the relationship between the two, further experiments were carried out in this study.

SWI/SNF is an ATP-dependent chromatin remodeling complex, and BRG1 is its core subunit [26]. Under physiological conditions, BRG1 is involved in regulating a variety of cellular processes, such as stem cell/progenitor cell self-renewal and response to hypoxia. In addition, the abnormal expression of BRG1 is related to the occurrence and development of many diseases [27]. Some studies have shown that the expression of BRG1 increases in the human liver fibrosis and liver fibrosis experimental model, and promotes the process of liver fibrosis by activating hepatic stellate cells [28]. Recent studies have found that BRG1 is a combined inhibitor of ZEB1. BRG1 and ZEB1 have a synergistic effect in inhibiting E-cad and initiating EMT [12]. It was also found that the inflammatory cell infiltration in the respiratory tract of asthmatic mice was related to the pro-inflammatory effect of BRG1, which was improved in BRG1 knockout mice [29]. In the in vitro EMT model of this study, the results indicated that BRG1 played a promoting role in the process of EMT. Dysfunctional BRG1 may be observed in some tumor tissues, but BRG1 may have different or even opposite effects, which is related to tissue specificity, the factors that interact with BRG1 and the functional activity of various proteins in SWI/SNF complex. For example, BRG1 may play a carcinogenic role in some tumors [30], and the increased expression of BRG1 is associated with tumor growth and invasiveness in prostate tumors [31]. Liu-ChengLi and JiaoQu found that uptake of exogenous HMGB1 (rhHMGB1) could induce changes in the expression of EMT markers in A549 and rat epithelial cells (RLE-6TN). Moreover, with the increase in rhHMGB1 concentration, the change in EMT markers was more obvious, and 48 h stimulated could promote the EMT process of cells more than 24 h stimulation [32]. Therefore, in this study, the optimal concentration of rhHMGB1 (4 ng/mL) was used to stimulate A549 cells. The results showed that after silencing the BRG1 gene, the EMT process was inhibited and the expression of HMGB1 was also inhibited, and the stimulation of rhHMGB1 could reverse this inhibitory effect. The results show that both HMGB1 and BRG1 play a promoting role in the process of EMT, and they interact with each other.

Some studies have pointed out that TGF-β1 can activate PI3K and then phosphorylate Akt, thus increasing protein synthesis in the process of EMT through the mTOR complex [33]. However, it is not clear whether HMGB1/BRG1 regulates the PI3K/Akt/mTOR pathway in the EMT process of A549 cells through these effectors. Some studies have shown that the activation of PI3K/Akt/mTOR signal pathway can promote the occurrence and development of pulmonary fibrosis [34]. Other studies have shown that when PI3K/Akt is inhibited, EMT is interfered, and the use of Akt inhibitors can partially reverse the EMT process [35]. Our results indicate that the PI3K/Akt/mTOR pathway is activated during the EMT process, while both HMGB1 and BRG1 have positive regulatory effects on the PI3K/Akt/mTOR pathway. In addition, the overexpression of α-SMA in pulmonary fibrosis is related to the activation of PI3K/Akt, and the interaction between TGF-β1 and PI3K/Akt promotes the formation of pulmonary fibrosis [36]. In addition, the activation of PI3K/Akt may be involved in the pathogenesis of pulmonary fibrosis by regulating its downstream molecules, such as mTOR and hypoxia-inducible factor-1 α [37]. It is precisely because of the important role of PI3K/Akt in regulating receptor-mediated signal transduction that targeting PI3K/Akt has become a new strategy for the treatment of pulmonary fibrosis.

HMGB1-targeting compounds have been used in pulmonary fibrosis models, but the clinical efficacy of these compounds remains to be determined. Anti-HMGB1 antibodies and HMGB1 neutralizing reagents, such as thrombomodulin, can reduce the level of HMGB1 in animals and reduce fibrotic lung injury [38]. In addition, as a natural compound, glycyrrhizic acid can directly bind to HMGB1 and inhibit the expression of HMGB1. Among the inhibitors targeting HMGB1, glycyrrhizic acid is the most widely used and the most effective, so this study chose glycyrrhizic acid as an intervention to explore its intervention effect and mechanism on experimental silicosis in mice. Studies have shown that HMGB1 exists in the early stages of pulmonary inflammation and late fibrosis, and glycyrrhizic acid inhibits the expression of HMGB1, which can slow down the progression of pulmonary inflammation and fibrosis [15]. Glycyrrhizic acid can not only inhibit SMAD2 and SMAD3 pathways mediated by TGF-β1 [39], but can also change the pathological morphology of the bile duct ligation model and prevent liver fibrosis [40]. In A549 and BEAS-2B cells, glycyrrhizic acid could reverse the EMT process of pulmonary epithelial cells and significantly reduce the migration ability of pulmonary epithelial cells by inhibiting HMGB1 [16], which is consistent with our results. However, the therapeutic effect and mechanism of glycyrrhizic acid on pulmonary fibrosis in SiO_2_-induced silicosis in mice have not been deeply studied, so we tried to explore these. 

In this study, the mouse silicosis model was established by non-exposed tracheal instillation. The intervention dose of glycyrrhizic acid in mice by Zhu, Chen, Jiang, and Tian et al. [41,42,43] varied in 1~200 mg/kg/d, and the intervention mode was tail vein, intragastric administration or intraperitoneal injection. The intervention dose for the chronic disease model was mostly fluctuated in 50 mg/kg/d, and mainly by intraperitoneal injection. Intraperitoneal injection has the least damage and the best intervention effect on mice, so in this study, glycyrrhizic acid was dissolved in a mixture of 2% DMSO + 98% and normal saline, and mice were injected intraperitoneally according to the daily dose of 50 mg/kg, and the same amount of solvent was injected intraperitoneally every day in the solvent control group. The mice were killed 28 days after the establishment of the model, and the changes of lung surface and texture were observed with naked eyes. It was found that compared with the control group, the lung texture of the SiO_2_ group was relatively hard, and small nodules could be seen on the surface. HE staining showed that the alveoli of mice were seriously injured and there were many inflammatory infiltration. Masson staining could see the proliferation of collagen fibers in lung tissue, which indicated that the experimental silicosis model in this study was successful. Compared with the SiO_2_ group and the DMSO group, the lung surface nodules of the Gly group were less and the texture was relatively soft, and the HE and Masson staining results showed that the lung injury of mice was milder, which indicated that glycyrrhizic acid could alleviate the symptoms of silicosis. Western blot and RT-qPCR further verified that compared with the SiO_2_ group and DMSO group, the collagen fibers in the Gly group were indeed less, the EMT process was inhibited, and the expression of HMGB1 and BRG1 were inhibited at varying degrees, which was consistent with the previous research results [44]. Considering that HMGB1 and BRG1 can act together on the PI3K/Akt/mTOR pathway in cell experiments, this study further verified in the animal model, found that the use of glycyrrhizic acid affects the expression of the PI3K/Akt/mTOR pathway, which is consistent with the trend in the EMT model in vitro, indicating that glycyrrhizic acid may inhibit the process of EMT in pulmonary fibrosis through the PI3K/Akt/mTOR pathway.

## 5. Conclusions

In summary, we found that HMGB1 interacts with BRG1 to promote the EMT process of pulmonary epithelial cells by activating PI3K/Akt/mTOR pathway. Glycyrrhizic acid can affect the BRG1 and PI3K/Akt/mTOR pathway by inhibiting the expression of HMGB1 and delay the progression of silicosis in mice.

## Data Availability

All data generated or analyzed during this study are included in this published article.

## Supplementary Materials

The following are available online at https://www.mdpi.com/article/10.3390/ijerph19148743/s1, Table S1: Human gene primer sequence; Table S2: Mouse gene primer sequence; Table S3: Source and dilution ratio of antibody.
