# Supplementary material for: Glycyrrhizic Acid Attenuates Pulmonary Fibrosis of Silicosis by Inhibiting the Interaction between HMGB1 and BRG1 through PI3K/Akt/mTOR Pathway
Source: Int J Environ Res Public Health. 2022 Jul 18;19(14):8743. doi: 10.3390/ijerph19148743 (PMC9317839; doi:10.3390/ijerph19148743)
Supplement: Supplementary file 1 [file ijerph-19-08743-s001.zip › ijerph-1782691-SI.pdf]

Table S1. Human gene primer sequence

Name	Sequence
GAPDH	Forward：5′- ACCCAGAAGACTGTGGATGG-3′
	Reverse：5′- TCTAGACGGCAGGTCAGGT-3′
E-cad	Forward：5′- AGGATGACACCCGGGACAAC-3′
	Reverse：5′- TGCAGCTGGCTCAAGTCAAAG-3′
N-cad	Forward：5′- CAGAATCGTGTCTCAGGCTCCAAG-3′
	Reverse：5′- CTGCGTTCCAGGCTGGTGTATG-3′
Vimentin	Forward：5′- CCTTGAACGCAAAGTGGAATC-3′
	Reverse：5′- GACATGCTGTTCCTGAATCTGAG-3′
α-SMA	Forward：5′- ATTGCCGACCGAATGCAGA-3′
	Reverse：5′- ATGGAGCCACCGATCCAGAC-3′
HMGB1	Forward：5′- GAAGAGGATGAGGAGGAGGAGGAAG-3′
	Reverse：5′- TACCACCAGGACAGGGCTATCTAAAG-3′
BRG1	Forward：5′- CAGATCCGTCACAGGCAAAAT-3′
	Reverse：5′- TCTCGATCCGCTCGTTCTCTT-3′

Table S2. Mouse gene primer sequence

Name	Sequence
GAPDH	Forward：5′- TCAGGAGAGTGTTTCCTCGT-3′
	Reverse：5′- TGCCGTGAGTGGAGTCATAC3′
E-cad	Forward：5′- CCTGTCTTCAACCCAAGCAC-3′
	Reverse：5′- CAACAACGAACTGCTGGTCA-3′
N-cad	Forward：5′- AGGACCCTTTCCTCAAGAGC-3′
	Reverse：5′- ATAATGAAGATGCCCGTTGG-3′
Vimentin	Forward：5′- CGGCTGCGAGAGAAATTGC-3′
	Reverse：5′- CCACTTTCCGTTCAAGGTCAAG-3′
α-SMA	Forward：5′- GAGCATCCGACACTGCTGAC-3′
	Reverse：5′- GCACAGCCTGAATAGCCACA-3′
HMGB1	Forward：5′- ATGAGGAAGAGGAGGAAGAAGAGGAAG-3′
	Reverse：5′- TACCACCAGGACAGGGCTATCTAAAG-3′
BRG1	Forward：5′- CACCTAACCTCACCAAGAAGATGA-3′
	Reverse：5′- CTTCTTGAAGTCCACAGGCTTTC-3′
PI3K	Forward：5′- ACACCACGGTTTGGACTATGG -3′
	Reverse：5′- GGCTACAGTAGTGGGCTTGG -3′
Akt	Forward：5′- TGGGTCAAGGAACAGAAGCA -3′
	Reverse：5′- TCACACTGACCACTGACACA -3′
mTOR	Forward：5′- CGGGACTCTTTACACTGCG -3′
	Reverse：5′- CCTTCAGGCTCAACCAACA -3′

Table S3. Source and dilution ratio of antibody

Name	Catalog Number	Source	Dilution
anti-Akt	Proteintech, 60203-2-Ig	Mouse	1:10000
anti-BRG1	abcam, ab110641	Rabbit	1:15000
anti-E-cadherin	Cell Signaling Technology, 3195	Rabbit	1:1000
anti-GAPDH	Proteintech, 60004-1-lg	Mouse	1:20000
anti-HMGB1	Proteintech, 10829-1-AP	Rabbit	1:1500
anti-mTOR	Proteintech, 66888-1-Ig	Mouse	1:10000
anti-N-cadherin	Servicebio, GB111009	Rabbit	1:1000
anti-phospho-Akt	Cell Signaling Technology, 4060	Rabbit	1:2000
anti-phospho-mTOR	Cell Signaling Technology, 5536T	Rabbit	1:1000
anti-phospho-PI3K	Cell Signaling Technology, 4228T	Rabbit	1:1000
anti-PI3K	Proteintech, 20584-1-AP	Rabbit	1:1000
anti-Vimentin	Servicebio, GB11192	Rabbit	1:1000
anti-α-SMA	Servicebio, GB13044	Rabbit	1:1500
